# The impact of an early intervention home-based program on body composition in preterm-born preschoolers with very low birth weight

**DOI:** 10.3389/fnut.2022.981818

**Published:** 2022-10-20

**Authors:** Rafael Oliveira Fernandes, Juliana Rombaldi Bernardi, Júlia Delgado da Fonseca, Franciéle Gomes da Silva, Renato Soibelmann Procianoy, Rita C. Silveira

**Affiliations:** ^1^Graduate Program in Child and Adolescent Health (PPGSCA), Medical School of Universidade Federal do Rio Grande do Sul (UFRGS), Hospital de Clínicas de Porto Alegre (HCPA), Porto Alegre, Brazil; ^2^Graduate Program in Food, Nutrition and Health, Medical School of Universidade Federal do Rio Grande do Sul (UFRGS), Hospital de Clínicas de Porto Alegre (HCPA), Porto Alegre, Brazil

**Keywords:** premature birth, very low birth weight (VLBW), early intervention, body composition, blood chemical analysis, preschool child

## Abstract

**Background and aims:**

Early child interventions focused on the family prevented neurodevelopmental and behavioral delays and can provide more knowledge regarding responsive feeding, thus creating learning opportunities to promote better quality nutrition and preventing failure to thrive. The aim is to verify the impact of a continuous program of early home-based intervention on the body composition of preschool infants who were born preterm with very low birth weight (VLBW).

**Methods:**

This is a longitudinal analysis from a randomized controlled trial, including VLBW preterm children, born in a tertiary hospital in Southern Brazil and followed up at the high-risk institutional ambulatory clinic. Participants were divided into the intervention group (IG): skin-to-skin care with the mother (kangaroo care), breastfeeding policy, and tactile-kinesthetic stimulation by mothers until hospital discharge. Subsequently, they received a program of early intervention with orientation and a total of 10 home visits, independently from the standard evaluation and care that was performed following the 18 months after birth; conventional group (CG): standard care according to the routine of the newborn intensive care unit (NICU), which includes kangaroo care, and attending to their needs in the follow-up program. Body composition estimation was performed using bioelectrical impedance analyses (BIA), and physical activity and feeding practices questionnaires were evaluated at preschool age, as well as anthropometric measurements and biochemical analysis.

**Results:**

Data of 41 children at 4.6 ± 0.5 years old were evaluated (CG *n* = 21 and IG *n* = 20). Body weight, height, body mass index, waist and arm circumferences, and triceps and subscapular skinfold did not differ between groups. The IG presented higher segmented fat-free mass (FFM) when compared to the CG (right arm FFM: 0.74 vs. 0.65 kg, *p* = 0.040; trunk FFM: 6.86 vs. 6.09 kg, *p* = 0.04; right leg FFM: 1.91 vs. 1.73 kg, *p* = 0.063). Interaction analyses showed that segmented FFM and FFM Index were associated with higher iron content in the IG. In the CG, interaction analyses showed that increased visceral fat area was associated with higher insulin resistance index.

**Conclusion:**

An early intervention protocol from NICU to a home-based program performed by the mothers of VLBW preterm children of low-income families presents a small effect on FFM.

## Introduction

Preterm birth is considered a health problem worldwide, with a higher incidence in low-income countries, with an average of 12%, compared to 9% in high-income countries (1). Despite preterm birth being the leading cause of death of children aged under 5 years, the improvement of neonatal care and follow-up programs to support this population is increasing survival rates, allowing more children to enter adulthood (2). Furthermore, preterm infants, mainly those born extremely preterm, present a higher incidence of developmental deficits (cognitive, motor, behavioral, communicative, learning, and sensory disorders) and a higher risk for delayed neurodevelopment, a condition that demands early implementation of multidisciplinary actions that may prevent negative outcomes (3, 4). Moreover, preterm infants present a higher risk for chronic and metabolic diseases with aging (5, 6).

Among the factors that increase their vulnerability to developing growth and developmental issues (5) is body composition, which has been associated with neurodevelopment in very low birth weight (VLBW) infants (7, 8). A higher rate of fat-free mass (FFM) was associated with improved cognitive and motor scores at 12 months of corrected age (9), and, on the other hand, a deficit of FFM was associated with neurological impairment in VLBW infants at 24 months of corrected age (10). Body composition studies have shown that preterm infants reach full-term equivalent age with less FFM and a higher percentage of total body fat (%TBF) when compared to their full-term equivalent counterparts (11, 12), as well as lower bone mineral density (13, 14) and increased abdominal adipose tissue (15). Since body growth and mineral accretion occur mainly in the last trimester of gestation, reduced muscle mass and skeletal mineralization could be related to preterm infants (12). Moreover, body fat and FFM gains in preterm infants are associated with several areas of cognitive function (16). Thus, early intervention, follow-up care for preventive action, and timely detection of possible adverse health outcomes are critical for preterm infants’ growth health.

The “first 1,000 days,” from conception to 24 months (17), are characterized as a window of opportunity to stimulate a child’s developmental domains, such as physical, language, cognitive, and social–emotional (18, 19). A systematic review showed that early intervention significantly affects child development, but it does not affect linear growth, which is more associated with nutritional intervention (20). The literature describes that nutritional intervention promotes short- and long-term health effects after preterm birth (21). Moreover, early tactile and kinesthetic stimulation in VLBW preterm children promoted a borderline higher psychomotor development and increased cognitive development assessed at 2 years corrected age, which did not affect weight, length, and head circumference (22). Early physical therapy intervention also presented a positive impact on VLBW preterm, hence reducing the incidence of motor delay (23). However, it is still not clear if an early intervention program could affect the body composition of the preterm population with advanced age. A single-blind cluster randomized controlled trial showed that early physical activity in the first months of life in term-born children promoted a reduced sum of skinfold, when compared to the non-stimulated group, without any differences in motor development (24).

There are few studies evaluating body composition in preschool VLBW preterm children subjected to early intervention and continuous clinical follow-up. Thus, our main goal is to investigate if a protocol of early home-based intervention program during the first 18 months of corrected age in VLBW preterm affected the body composition once they reach preschool age, comparing them with a group subjected to conventional care protocol. Also, this study investigated if the body composition results in response to the intervention protocol were related to neonatal, growth, and biochemical characteristics evaluated during these follow-ups.

## Materials and methods

### Design and study population

This was a longitudinal analysis from a randomized controlled trial that investigated preschool VLBW preterm children that were subjected to an early, continuous, and global intervention with a parent’s orientation program in the first 12–18 months of corrected age. This program was a randomized clinical trial (RCT), performed from 2016 to 2019, previously described in the study protocol (4), with children born in the Hospital de Clínicas de Porto Alegre (HCPA), a level-3 referral center for high-risk neonates in South Brazil, with regular follow-up until 5 years of age. The study population was preterm children born at less than 32 weeks of gestational age (GA) or children of birth weight less than 1,500 g during the 48 h after childbirth. Newborns with a major congenital malformation or inborn errors of metabolism, STORCH complex infections, HIV, or autoimmune conditions were excluded.

**FIGURE 1:**
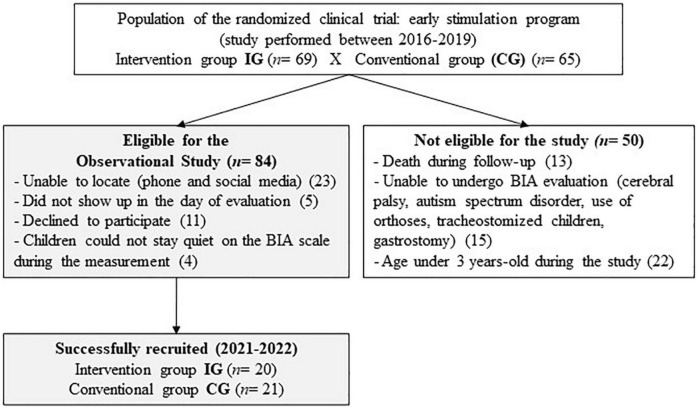
Flowchart.

For the follow-up study, the parents were invited to accompany their children, aged 3 and 5 years, to perform a body composition analysis (Figure 1). Exclusion criteria for this investigation were children that died before reaching preschool age, children with severe motor, cognitive, or organic sequelae that prevented the use of bioimpedance scale (cerebral palsy, autism spectrum disorder, use of orthoses, tracheostomized children, and gastrostomy), and children who did not complete 3 years of age during the study period. The recruitment was performed by phone calls and use of social media to locate the parents with no updated phone numbers in the medical records.

The preschool children evaluated in this study were those who were randomized to one of the two groups from the previous clinical trial (4). The intervention group (IG) was characterized by skin-to-skin care (kangaroo care), breastfeeding policy, and massage therapy made by the mothers until hospital discharge. After discharge, mothers received orientation for continuous global stimulation at home plus 10 home visits by the research team, regardless of the standard evaluation and care provided in the follow-up clinic to all preterm children. The systematic early intervention program was based on developmental milestones, anticipating by a month the evolutionary step acquisition of motor and/or cognitive expected for corrected age. The conventional group (CG) was characterized as receiving standard care according to the routine care of the newborn intensive care unit (NICU) and according to the subject’s needs in the follow-up program. The follow-up program used in this study is an extension of neonatal and perinatal care, in which the research team provides conditions to monitor growth, development, and common morbidities with a multidisciplinary team who can fully assess the child and the caregivers, parents, family members, and the school.

### Outcome measures

#### Physical exam during newborn intensive care unit and follow-up at an institutional ambulatory clinic

Physical exams for measurements of weight (kg), height (cm), head circumference (cm), and body mass index (BMI, kg/m^2^) were performed at birth, at NICU discharge, and at monthly and annual assessments as a routine appointment at the ambulatory clinic until the appointment for bioelectrical impedance analysis (BIA). All measurements in the ambulatory clinic were performed by a Neonatologist, using standard techniques and calibrated instruments (electronic scale and stadiometer). Z-scores of weight-for-age (W/A), length-for-age (L/A), and BMI-for-age (BMI/A) were evaluated using the *Anthro*^®^ software (25), considering overweight: BMI/A *Z*-score > + 1, obesity: BMI/A *Z*-score > + 2, and underweight: BMI/A *Z*-score < –2.

At the evaluation appointment for BIA in the preschool-age children, all anthropometric measurements were performed in duplicate for which the average results were considered: waist circumference (cm) measured with an inelastic measuring tape (in cm) placed at the umbilical scar level at the end of the child’s exhale in orthostatic position; arm circumference (cm), measured in the mid-point of the upper arm; triceps skinfold thickness (mm), measured by the midpoint between the acromion and the olecranon; and, subscapular skinfold thickness (mm), measured diagonally below the inferior angle of the scapula. Skinfold measurements (triceps and subscapular) were assessed using a skinfold caliper (*Lange*^®^, Ann Arbor, Michigan, USA).

#### Socioeconomic status

Data related to the mother’s schooling level (in years) and household income (in BRL per month) were assessed on the day of the follow-up appointment when participants reached preschool age, on the same day of BIA.

#### Neonatal and follow-up data

Neonatal information from the NICU stay, follow-ups, and clinical appointments was collected from in-hospital patient records. During the NICU period, maternal variables were collected (maternal age, preeclampsia, gestational diabetes, and use of corticosteroid). The neonatal variables were gestational age (GA—evaluated by last menstrual period and confirmed by early obstetrical ultrasound and neonatal clinical examination), birth weight, gender, the status of small for gestational age (SGA; defined as birth weight < 10th percentile) (26), 5-min Apgar score, length of hospital stay, necrotizing enterocolitis, central nervous system injury (periventricular hemorrhage and/or leukomalacia), bronchopulmonary dysplasia as oxygenotherapy at 36 weeks corrected age, retinopathy of prematurity, and packed red blood cell transfusion. Anthropometric measures were collected at NICU discharge.

In addition to physical exams in the follow-up clinic, information regarding breastfeeding practices was examined and classified as exclusive breastfeeding, exclusive infant formula, or combination feeding. Exclusive breastfeeding is the action of feeding solely with human milk coming from the mother’s breast, with no other liquids or solids (27).

A biochemical exam was also assessed in the records in the follow-up consultation at the 24th month of corrected age. Venous blood sampling (12 h fasting) was collected for routine blood tests according to the follow-up program for measuring hemogram; serum iron (μg/dL); total iron binding capacity (μg/dL); ferritin and transferrin (mg/dL); total cholesterol; LDL-cholesterol; HDL-cholesterol; non-HDL cholesterol and triglycerides (mg/dL); glucose (mg/dL); insulin (μU/mL); and cortisol (μg/dL). Dyslipidemia was determined when one or more lipid markers presented values, such as total cholesterol > 200 mg/dl; LDL-cholesterol > 130 mg/dL; HDL-cholesterol < 40 mg/dL; non-HDL cholesterol > 145 mg/dL; and triglycerides > 100 mg/dL (28). The insulin resistance index was calculated as a homeostatic model assessment for insulin resistance (HOMA-IR) [(glucose (mg/dL) × insulin (μU/mL))/405].

#### Body composition, feeding practices, and physical activity at preschool age

Body composition was evaluated using a multi-frequency bioelectrical impedance analysis (BIA) using InBody 770^®^ (Biospace, South Korea), a tetrapolar electrode configuration system. Parents were instructed to fast their children for at least 3 h before the measurement, and diapers were changed before children got up on the scale. Each participant was positioned in an orthostatic position on a platform with lower electrodes for the feet and the hands holding onto upper electrodes, in which children were to hold this position for 1 min until completion of the measurement. This evaluation accurately measures body weight (BW in kg), the body water content in liters (total body water (TBW), water inside and outside cells, and water in the segments), fat mass (FM; in kg), total body fat (%TBF), fat-free mass (FFM = BW–fat mass, in kg; segmented FFM of arms, legs, and trunk—the head is not considered in the segmented measurements), FFM Index (FFMI, in kg/m^2^), lean mass (LM = water + proteins + non-osseous mineral; in kg), skeletal muscle mass (in kg), proteins and minerals (in kg), bone mineral content (BMC = osseous mineral; in kg), visceral fat area (in cm^2^), cellular body mass (in kg), arm circumference (in cm), and basal metabolic rate (BMR, in kCal).

Physical activity levels were measured using a structured questionnaire that estimates the sedentary and active time of children, considering activities on weekdays or weekends and day shifts (morning, afternoon, and night), presented as total hours of activity per week. The measure of physical activity is expressed by the daily time of participation in games and outdoor play while the measure of sedentary behavior is based on the time spent watching television.

### Data analysis

Descriptive statistics are shown as mean with standard deviation (± SD) or median with interquartile range for continuous variables, according to the Shapiro-Wilk normality test, and counts with proportions for categorical variables. Between-group comparisons were performed with Student’s *t*-test, Mann-Whitney *U*-test, Pearson’s Chi-squared test, or Fisher’s exact test. Cohen’s *d* was used to measure effect size for quantitative variables. The power to compare the FFM segment averages between conventional and intervention groups was calculated considering a 5% significance level and the sample size of this study (IG = 20 and CG = 21). First, the relationship between neonatal data (GA, birth weight, and head circumference), physical exam results at 12 months (body weight, height, and BMI/A), and biochemical variables with body composition were explored (TBW, FM, FFM, and BMC) by Pearson’ or Spearman’ correlation (a hypothesis-generating analyses). Analysis of covariance (ANCOVA) was performed to compare body composition outcomes between groups and to analyze the relationship between them and the biochemical variables. They were adjusted for gender due to literature differences between outcomes by gender (29–31). *P*-values under 0.05 were considered statistically significant. All analyses were performed using the SPSS (Statistical Package for the Social Science) Program Version 18.0 (IBM SPSS Statistical for Windows, Armonk, New York, USA).

## Results

Our investigation evaluated 41 VLBW preterm children at preschool age who participated previously in a clinical trial of an early intervention program developed from February 2016 to December 2019. Briefly, of the 134 children from the previous clinical trial, 84 were eligible to participate in this new investigation (Figure 1). For the current study, BIA and physical exam results were collected from December 2020 to June 2022 from the children whose guardians have accepted to participate.

Table 1 shows the characteristics of infants of the general sample population, as well as their respective groups, CG (*n* = 21) and IG (*n* = 20). The mean age of children when BIA was performed was 4.7 ± 0.5 years, 17 (41%) girls and 24 (59%) boys. There were no differences in anthropometric variables, such as body weight, height, waist and arm circumferences, and skinfold measurements from the children of both groups. Overall, 22% of the sample were classified as overweight or obese and 7% as underweight, without any significant differences between groups. Maternal schooling level and family income did not differ between groups. Regarding neonatal data of the preterm children randomly assigned in each group, the mean gestational age was 28 ± 2 weeks, birth weight 1,073 ± 318 g, head circumference 25.5 ± 2.4 cm, and 4 (11%) were SGA without statistical differences between groups. Also, neonatal comorbidities were not statistically different between groups. In both groups, birth weight correlated positively with TBW (IG: *r* = 0.462, *p* = 0.041; CG: *r* = 0.501, *p* = 0.021) and with FFM (IG: *r* = 0.461, *p* = 0.041; CG: *r* = 0.487, *p* < 0.025). Birth weight also correlated positively with FM only in CG: *r* = 0.472, *p* = 0.031 (IG *r* = 0.289, *p* = 0.22), and no significant association with BMC was observed. GA did not present a correlation with TBW, FFM, FM, and BMC when analyzed in each group separately (*p* > 0.05).

**TABLE 1:** Current and neonatal characteristics of preterm children subjected to the early intervention program compared to conventional care.

Characteristic	General (*n* = 41)	Conventional (*n* = 21)	Intervention (*n* = 20)	*P*-value	Cohen-*d*
**Current period (preschoolers)**					
Age at examination, years	4.6 ± 0.5	4.5 ± 0.5	4.7 ± 0.5	0.758	0.40
Female/male, *n* (%)	17 (41)/24 (58)	9 (43)/12 (57)	8 (40)/12 (60)	0.602	–
Weight, Kg	18.1 ± 4.0	17.4 ± 3.3	18.8 ± 4.6	0.481	0.35
Weight-for-age, *Z*-score	0.03 ± 1.54	–0.10 ± 1.46	0.17 ± 1.65	0.762	0.17
Height, m	1.05 ± 0.06	1.04 ± 0.06	1.06 ± 0.06	0.522	0.33
Length-for-age, *Z*-score	–0.39 ± 1.25	–0.49 ± 1.35	–0.29 ± 1.16	0.666	0.16
Body mass index (BMI), Kg/m^2^	15.6 (14.6–17.7)	15.6 (14.5–17.2)	15.2 (14.7–18.9)	0.824	–
BMI-for-age, *Z*-score	0.43 ± 1.59	0.33 ± 1.18	0.52 ± 1.96	0.946	0.12
Overweight/obesity, *n* (%)	9 (22)	3 (14)	6 (30)		–
Underweight, *n* (%)	3 (7)	0 (0)	3 (2)		–
Waist circumference, cm	51.5 (47–55)	52 (48–53.9)	51.5 (47.5–58.5)	0.686	–
Arm circumference, cm	16.9 ± 2.6	16.3 ± 2.3	17.5 ± 2.7	0.096	0.48
Triceps fold, mm	8.5 (6.8–11.0)	8.0 (6.0–11.3)	9.5 (7.0–10.5)	0.370	–
Subscapular fold, mm	5.6 (4.0–7.2)	5.0 (4.0–6.0)	6.5 (4.3–8.0)	0.212	–
**Current socioeconomic status**					
Maternal education, years	11.1 ± 3.12	10.9 ± 3.1	11.3 ± 2.8	0.719	0.13
Family income, Brazilian R$ (×1000)	2.9 (1.5–3.8)	3.0 (1.2–3.8)	2.5 (1.5–4.6)	0.694	–
**Neonatal period**					
Gestational age (GA), weeks	28.1 ± 2.0	28.0 ± 1.8	28.2 ± 2.2	0.701	0.10
Birth weight, g	1,073 ± 318	1,175 ± 308	1,172 ± 335	1.000	0.00
Birth length, cm	36.0 (33.5–39.7)	35.5 (33–39.7)	37.0 (34–39.7)	0.551	–
Birth head circumference, cm	25.5 ± 2.4	25.7 ± 2.2	25.4 ± 2.6	0.739	0.12
Small for GA (SGA), *n* (%)	4 (10.8)	2 (10)	2 (11)	0.298	–
Apgar score 5 min	8 (7–9)	8 (6–8)	8 (7–9)	0.312	–
Maternal age, years	31 (23–35)	33 (29–36)	28.5 (19–31)	0.009	–
Pre-eclampsia, *n* (%)	16 (39)	8 (38)	8 (40)	0.901	–
Gestational DM (GDM), *n* (%)	2 (5)	1 (5)	1 (5)	0.972	–
Antenatal corticosteroids, *n* (%)	37 (90)	21 (100)	16 (80)	0.031	–
Use of surfactant, *n* (%)	25 (61)	14 (67)	11 (55)	0.444	–
BPD, *n* (%)	13 (32)	5 (24)	8 (42)	0.217	–
Parenteral nutrition (PTN), days	15 (11–23)	14 (11–25)	15 (10–18)	0.539	–
Full enteral feeding, days	23 (12–30)	16 (12–28)	18 (15–30)	0.428	–
Necrotizing enterocolitis, *n* (%)	5 (12)	2 (9)	3 (15)	0.881	–
Lesion CNS, *n* (%)	18 (44)	9 (42)	9 (45)	0.890	–
PIVH, *n* (%)	16 (39)	8 (38)	8 (40)	0.901	–
Leukomalacia, *n* (%)	3 (5.6)	2 (9)	1 (5)	0.935	–
Ductus arteriosus, *n* (%)	19 (46)	10 (47)	9 (39)	0.867	–
Ibuprofen, *n* (%)	16 (39)	10 (42)	6 (30)	0.248	–
ROP, *n* (%)	11 (27)	5 (24)	6 (30)	0.655	–
Packed RBC transfusion	2 (0–4)	1 (0–4)	2 (0–4)	0.894	–
NICU stay, days	64 (52–99)	64 (55–100)	59 (49–100)	0.382	–
Body weight at discharge, g	2,691 ± 544	2,764 ± 660	2,615 ± 390	0.387	0.27
Length at discharge, cm	45.5 ± 2.8	46.0 ± 3.0	45.0 ± 2.4	0.402	0.36
Head circumference at discharge, cm	33.4 ± 1.6	33.4 ± 2.0	33.3 ± 1.1	0.810	0.06

Data presented as mean ± SD, Median (P25–75), or absolute number (*n*) and proportion (%).

Parametric variables: independent sample *t*-test.

Non-parametric variables: Mann-Whitney *U*-test. Categorical variable: Chi-square test.

BMI, Body Mass Index; GDM, Gestational diabetes Mellitus; BPD, bronchopulmonary dysplasia; CNS, Central Nervous System; PIVH, peri intraventricular hemorrhage; ROP, Retinopathy of prematurity; RBC, red blood cells; NICU, neonatal intensive care unit.

Physical examination performed during the first follow-up appointment after NICU discharge and 12 months corrected age did not show a statistical difference between groups. The nutrition offered to the preterm children in the first weeks of life at home did not vary between groups (Table 2). Biochemical analyses performed at 24 months of corrected age did not show statistical differences among groups (Table 3).

**TABLE 2:** Physical exam in the first ambulatory follow-up visit after NICU discharge and 12 months corrected age of preterm children subjected to early intervention program compared to conventional care.

Characteristic	General	Conventional	Intervention	*P*-value	Cohen-*d*
**First ambulatory appointment after discharge**	***n* = 39**	***n* = 20**	***n* = 19**		
Chronological age, weeks	13 ± 5	15 ± 5	12 ± 4	0.133	–
Body weight, kg	3.06 (2.74–3.86)	3.55 (2.65–4.53)	2.96 (2.74–3.45)	0.175	–
Length, cm	48 (46–51)	50 (47–54)	47 (46–5,149)	0.063	–
Cephalic perimeter, cm	35.9 ± 2.4	36.7 ± 2.8	35.2 ± 1.7	0.058	0.65
Body Mass Index, kg/m^2^	13.7 ± 2.0	14.0 ± 2.2	13.5 ± 1.8	0.429	0.25
Breast milk, *n* (%)	4 (10)	2 (10)	2 (10)		–
Combination feeding, *n* (%)	19 (46)	12 (60)	7 (37)		
Infant formula, *n* (%)	16 (39)	6 (30)	10 (53)	0.319*	
**Ambulatory appointment at 12 months of corrected age**	***n* = 37**	***n* = 20**	***n* = 17**		
Chronological age, months	15 ± 1	15 ± 1	15 ± 1	0.361	–
Body weight, kg	9.03 ± 1.68	9.29 ± 1.59	8.73 ± 1.64	0.304	0.34
Length, cm	74 ± 4	75 ± 4	73 ± 4	0.357	0.50
Cephalic perimeter, cim	45.7 ± 1.9	46.1 ± 1.7	45.4 ± 2.1	0.266	0.37
Body mass index, kg/m^2^	16.1 ± 1.5	16.3 ± 1.5	15.9 ± 1.5	0.374	0.27

Data presented as mean ± SD, Median (P25–75), or absolute number (*n*) and proportion (%).

Parametric variables: independent sample *t*-test. Non-parametric variables: Mann-Whitney *U*-test. Categorical variable: Chi-square or Fisher’s exact test*.

**TABLE 3:** Biochemical analysis in the follow-up ambulatory care at 24 months corrected the age of preterm infants subjected to an early intervention program compared to conventional care.

Biochemical parameter	General (*n* = 31)	Conventional (*n* = 15)	Intervention (*n* = 16)	*P*-value	Cohen-*d*
Hemoglobin, g/dL	12.6 ± 1.1	12.4 ± 1.0	12.7 ± 1.2	0.615	0.27
Hematocrit,%	36.8 ± 3.2	36.6 ± 3.1	37.0 ± 3.5	0.792	0.12
Serum iron, μg/dL	83.4 ± 25.1	77.3 ± 24.8	89.2 ± 24.8	0.192	0.48
Ferritin, mg/dL	33.2 (23.4–55.8)	25 (21.4–50.7)	37.5 (24.0–54.4)	0.257	–
Transferrin, mg/dL	282 ± 45	287 ± 52	278 ± 40	0.644	0.19
Transferrin saturation, %	24.1 ± 8.1	23.0 ± 8.3	25.3 ± 8.17	0.459	0.28
Total iron binding capacity, μg/dL	252 (226–305)	258 (222–309)	251 (226–312)	0.949	–
Glucose, mg/dL	86.5 ± 8.6	86.9 ± 9.6	86.0 ± 7.8	0.789	0.10
Insulin, μU/mL	2.9 (2.3–4.1)	2.6 (1.8–3.6)	3.9 (2.5–5.0)	0.101	–
HOMA-IR	0.74 ± 0.45	0.59 ± 0.24	0.89 ± 0.56	0.107	0.69
Cortisol, μg/dL	9.8 ± 4.6	9.5 ± 5.8	10.0 ± 3.2	0.775	0.11
Total cholesterol, mg/dL	143 ± 26	141 ± 23	145 ± 30	0.709	0.15
LDL-cholesterol, mg/dL	85 ± 30	85 ± 22	85 ± 37	0.995	0.00
HDL-cholesterol, mg/dL	43 ± 11	39 ± 8	46 ± 12	0.084	0.68
Triglycerides, mg/dL	89 (66–133)	71 (61–99)	114 (86–170)	0.074	–
Dyslipidemia, *n* (%)	19 (65)	10 (71)	9 (60)	0.782	–

Data presented as mean ± S.D., Median (P25–75), or absolute number (*n*) and proportion (%).

Parametric variables: independent sample *t*-test. Non-parametric variables: Mann-Whitney *U*-test.

HOMA-IR, homeostatic model assessment for insulin resistance.

Table 4 shows the results of the BIA in preschool age. FM and %TBF did not differ significantly between CG and IG groups. Although the IG presented a non-significant increase in total FFM, FFMI, and skeletal muscle mass when compared to the GC group, they demonstrated a significant increase in segmented FFM in the children subjected to early intervention (right arm (*p* = 0.040), left arm (*p* = 0.053), trunk (*p* = 0.040), and an overall tendency in right leg (*p* = 0.063) and left leg (*p* = 0.054). The statistical power to test if there were a minimal difference of 0.09 kg in the mean of right arm FFM between groups was 57%. For the means of trunk FFM, a power of 53% was calculated, considering a minimal difference of 0.77 kg between groups. Moreover, the means of left leg FFM yielded a 51% power, considering a minimal difference of 0.19 kg among groups. These power values were obtained considering a 5% statistical level. TBW content also showed an increased pattern, as observed in the FFM from IG, although not significant. BMC, BMR, and visceral fat area were similar between groups. Finally, we observed that TBW/FFM ratio in preterm children was 74.3% (95% CI 74.0–74.5).

**TABLE 4:** Body composition in preterm infants subjected to early intervention program compared to conventional care.

Body composition analysis	General (*n* = 41)	Conventional (*n* = 21)	Intervention (*n* = 20)	*P*-value	Cohen-*d*
Total body water (TBW), liters	10.7 ± 1.6	10.4 ± 1.3	11.1 ± 1.7	0.125	0.47
Intracellular water, liters	6.6 ± 1.01	6.4 ± 0.8	6.9 ± 1.1	0.110	0.52
Extracellular water, liters	4.1 ± 0.6	3.9 ± 0.5	4.2 ± 0.6	0.166	0.54
Fat mass (FM), Kg	2.7 (1.9–5.4)	2.7 (2.0–4.7)	3.0 (1.5–5.7)	0.938	–
Total body fat (TBF%)	16.3 (12.4–26.7)	16.3 (13.1–25.1)	17.6 (9.2–17.5)	0.657	–
Fat mass Index (FMI), kg/m^2^	2.6 (1.8–4.8)	2.4 (1.9–4.2)	2.6 (1.3–5.2)	0.804	–
Fat-free mass (FFM), Kg	14.5 ± 2.1	14.0 ± 1.9	15.0 ± 2.3	0.146	0.48
FFM Index (FFMI), Kg/m^2^	12.9 ± 0.8	12.8 ± 0.6	13.1 ± 0.9	0.156	0.39
Bone mineral content, Kg	0.68 ± 0.15	0.67 ± 0.15	0.69 ± 0.14	0.741	0.14
Lean mass, Kg	13.8 ± 2.0	13.3 ± 1.7	14.3 ± 2.2	0.126	0.51
Skeletal muscle mass, Kg	6.7 ± 1.3	6.3 ± 1.1	7.0 ± 1.4	0.098	0.56
Protein, Kg	2.86 ± 0.43	2.75 ± 0.36	2.98 ± 0.48	0.096	0.55
Minerals, Kg	0.87 ± 0.18	0.86 ± 0.19	0.87 ± 0.18	0.954	0.05
Segmented FFM					
FFM of right arm, Kg	0.70 ± 0.13	0.65 ± 0.09	0.74 ± 0.16	0.040	0.70
FFM of left arm, Kg	0.68 ± 0.14	0.66 ± 0.10	0.74 ± 0.16	0.053	0.61
FFM of trunk, Kg	6.46 ± 1.21	6.09 ± 0.90	6.86 ± 1.39	0.040	0.67
FFM of right leg, Kg	1.82 ± 0.30	1.73 ± 0.23	1.91 ± 0.35	0.063	0.61
FFM of left leg, Kg	1.81 ± 0.31	1.72 ± 0.24	1.91 ± 0.35	0.054	0.64
Segmented water content					
Right arm water, liters	0.54 ± 0.10	0.51 ± 0.07	0.58 ± 0.12	0.062	0.72
Left arm water, liters	0.54 ± 0.11	0.51 ± 0.08	0.58 ± 0.13	0.066	0.67
Trunk water, liters	5.04 ± 0.94	4.75 ± 0.72	5.33 ± 1.06	0.053	0.65
Right leg water, liters	1.41 ± 0.24	1.35 ± 0.18	1.48 ± 0.27	0.075	0.57
Left leg water, liters	1.41 ± 0.24	1.34 ± 0.19	1.49 ± 0.27	0.066	0.65
Additional data					
TBW/FFM, %	74.3 (73.9–74.5)	74.1 (73.8–74.6)	74.2 (74.1–74.5)	0.440	–
Basal metabolic rate, Kcal	686 ± 49	677 ± 47	695 ± 51	0.255	0.37
Visceral fat area, cm^2^	13.7 (9–17.1)	13.4 (9.3–16.0)	13.9 (8.6–18.7)	0.705	–
Cellular body mass, Kg	9.5 ± 1.4	9.2 ± 1.2	9.9 ± 1.5	0.098	0.52
Arm circumference, cm	19.2 ± 2.6	18.8 ± 2.06	19.7 ± 3.1	0.322	0.35
Arm muscle circumference, cm	15.2 ± 1.9	14.9 ± 1.4	15.6 ± 2.3	0.281	0.37
Full body phase angle (50 kHz)	4.6 (4.3–4.8)	4.4 (4.1–4.6)	4.7 (4.4–5.3)	0.111	–

Data presented as mean ± S.D., Median (P25–75), or absolute number (*n*) and proportion (%).

Parametric variable: independent sample *t*-test. Non-parametric variable: Mann-Whitney *U*-test.

TBW/FFM ratio, Total Body Water/Fat-free mass.

Regarding physical activity, total active hours during the week were significantly higher in CG (median 16 h (14–19.5) when compared to IG [10 h (6–18); *p* = 0.015)] (data not shown).

**FIGURE 2:**
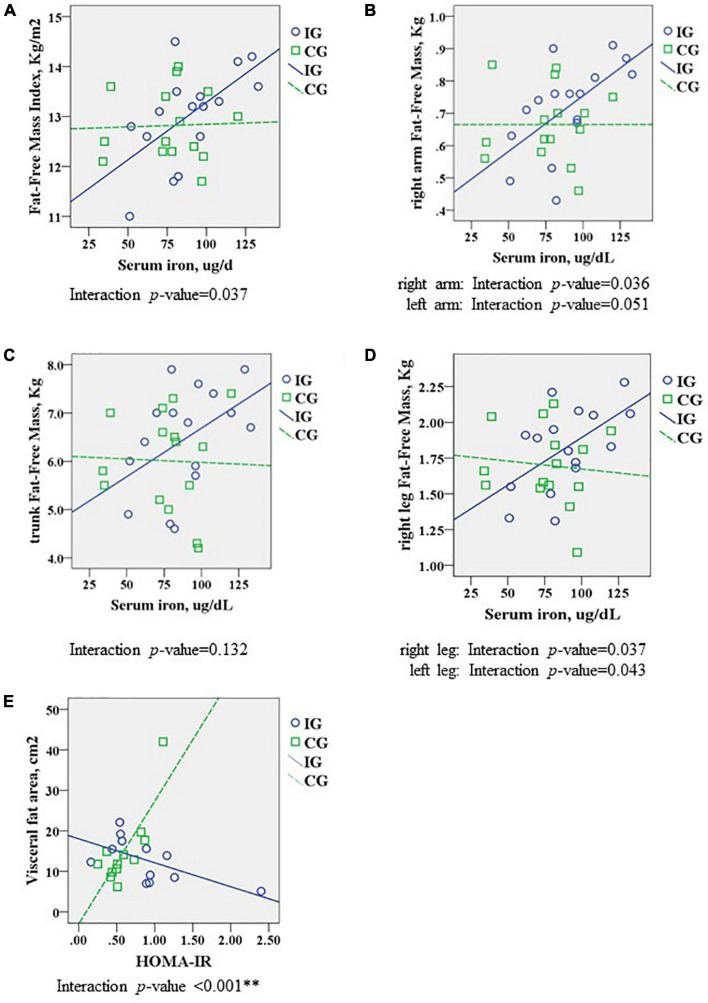
Comparison of body composition analysis at preschool age between interventional group (IG) and conventional group (CG), analyzing the interaction with biochemical blood results (blood exam at 24 months of corrected age), adjusted by gender. Association of **(A)** FFM Index and serum iron, **(B)** right arm FFM and serum iron, **(C)** trunk FFM and serum iron, **(D)** right leg FFM and serum iron, and **(E)** visceral fat area and HOMA-IR. Data were analyzed using ANCOVA, presenting interaction significant with a *p*-value < 0.05. FFM, fat-free mass; HOMA-IR, homeostatic model assessment for insulin resistance. ^**^Removing the two outliers, interaction is still significant (*p* = 0.005).

Analyses of interaction were performed to investigate if the association between body composition and biochemical analyses differed among groups. Overall, Figure 2 shows that FFM with iron content differed between IG and CG, even after adjusting by gender. IG group showed that FFMI increased by 0.26 kg/m^2^ (95%CI: 0.002–0.049; *p* = 0.037) per unit of increased serum iron, but no increase was observed in CG. Moreover, IG showed an increase of segmented FFM per unit of iron content (adjusted by gender): right arm: 0.004 kg (95%CI: 0.000–0.008; *p* = 0.036), left arm 0.004 kg (95%CI: 0.000–0.008; *p* = 0.051), trunk 0.025 kg (95%CI: 0.008–0.058; *p* = 0.132), right leg 0.009 kg (95%CI: 001–0.017; *p* = 0.037), and left leg 0.009 kg (95%CI: 0.00–0.017; *p* = 0.043) per unit of iron content. Visceral fat area interaction significantly decreased to 37 cm^2^ (95%CI: –50 to –25; *p* < 0.001) per unit of HOMA-IR in IG, an opposite response when compared to CG (Figure 2E) (removing the two outliers, interaction is still significant, *p* = 0.005^**^).

There was no significant interaction between the FFMI and segmented FFM with physical activity when adjusted by gender. Also, no association was established between feeding practices and FFM (data not shown).

## Discussion

For this study, we investigated the body composition of preterm VLBW children at preschool age who were subjected to a protocol of skin-to-skin care and global stimulation in a home-based program for 18 months. This investigation observed that early intervention may increase FFM in the body segments. On the other hand, despite stimulation implementation and closer family monitoring, no change in fat mass was observed. Nonetheless, this protocol may present a positive effect on reducing the relationship between visceral fat mass and insulin resistance.

Early life exposures to certain environmental factors during critical periods of development and growth may have significant short- and long-term consequences on an individual’s health: for that reason, early intervention is a strategy to improve growth and developmental outcomes (32, 33). In preterm infants, studies have shown that early intervention can improve cognition, increases growth and global development (34, 35), and attenuate the decrease in bone strength that may reduce the risk of osteopenia (36). Moreover, it presents a positive effect on motor skills through environmental enrichment (37). However, there are few early interventions implemented in the “first 1,000 days” and limited data on these effects in VLBW children regarding body composition across childhood.

Growth assessment is generally based on anthropometric measurements, which gives insufficient attention to growth quality. Thus, the assessment of body composition through BIA provides additional information on the relationship between growth and development (12). In a cohort study that compared extremely preterm children with full-term, in whose body composition was measured by DEXA, it was observed that preterm children presented the same height and weight as full-term. However, the preterm group presented lower values for muscle mass [0.9 kg (95%CI: 0.3–1.5)], total bone mineral density *z*-score [0.30 units (95%CI: 0.13–0.52)] and fat mass ratio [0.14 units (95%CI 0.06–0.21)] (13). In our study, the early stimulation protocol was performed anticipating, by a month, the motor and cognitive acquisition steps expected for corrected age, approaching near-term development; the usual growth evaluations (BMI, body circumferences, and skinfolds) did not present differences over the first year between intervention and conventional groups. Nonetheless, BIA showed that the early intervention program caused a small, but significant increase of segmented FFM, a body component comprised of skeletal muscle mass, body cell mass, total body water, connective tissue, and bone mineral mass. The use of a multi-frequency BIA provides a more direct portrayal of water compartments, increasing results reliability (38). Skeletal muscle accounts for a large proportion of the FFM, and it is known that physical activity positively affects FFM accretion from birth onward (39). Loss of muscle mass is associated with poor prognosis, reduced quality of life, and increased mortality, thus highlighting the muscle as an important component of whole-body metabolism, glucose homeostasis, as well as overall health and wellbeing (40). A systematic review showed that preterm infants present less lean tissue but a similar fat mass than full-term infants (11). Muscle growth can be activated by mechanical, oxidative, and energetic distress, and influenced by the availability of nutrients, growth factors, and cytokines (41). Moreover, an experimental study showed that mechano-signaling pathways stimulated by passive movements can control myofibrillar protein synthesis (42). Our finding showed that early intervention increased FFM, corroborating a previous investigation in which motor physical therapy in preterm in NICU increased lean mass (43). The preterm infants from the intervention group presented more FFM, despite performing less physical activity during the week compared to CG. The evaluation was done by physical questionnaire and supported our hypothesis that early intervention may contribute to an increase in the components of FFM. Moreover, an observational study showed that the gain of FFM in the first 4 years of the life of preterm children was associated with higher full-scale IQ and processing speed performance, which may enhance preschool cognitive performance (16).

Evidence from the classical birth cohorts from Pelotas, South Brazil showed that prematurity was associated with decreased total body fat and FFM, but with higher fat mass in adulthood (in male) (44). Higher fat mass is associated with an increased risk for metabolic syndrome in the preterm population (45). The early intervention protocol did not impact fat mass or bone mineral content within our intervention group. The metabolic bone mineral disease of prematurity is highly prevalent in VLBW preterm as it may occur due to loss of mineral transfer from the placental in the latest trimester and the reduced mechanical stimulation from the fetus against the uterine wall. This deficiency could be prevented by minerals and vitamin D intake, as well as physical activity (46–48). We did not observe changes in bone mineral content in response to early intervention protocol. However, a study with motor stimulation in preterm infants (26–34 weeks), with birth weight < 1,600 g, was able to increase bone mineral content evaluated by DEXA (43). A clinical study with extreme preterm-born young adults presented reduced area bone mineral density when compared to sex- and age-matched full-term controls, showing the long-term consequences of bone health (14).

Biochemical analysis performed in the follow-up clinic, at 24 months of corrected age, may support the structural findings observed in response to the early stimulation protocol. A significant relationship between iron content and FFM was observed only in IG, suggesting that the mechanical and global stimulus positively affects the components of FFM, such as the skeletal muscle which is increased by 700 g on average when compared to the CG group. Iron is an essential component of hemoglobin and myoglobin, in which iron supports muscle metabolism and healthy connective tissue, and is essential for physical growth, neurological development, and cellular function (49). Since iron is a micronutrient necessary for early development, it could be postulated that implementing an early intervention protocol of 18 months could improve muscle function, thus promoting iron homeostasis in the muscle system (50). Although no difference was observed in iron content between groups, a recent experimental study of a neurological disease demonstrated that regular physical exercise modulates iron homeostasis, in which dysregulation of iron metabolism leads to pathophysiological pathways (51).

Clinical studies indicate that preterm individuals have physiological disease pathways that differ from those born at full-term (52). In this same context of different mechanisms between preterm and full-term individuals, we observed that the hydration factor (TBW/FFM) in our preschool preterm children was 74.3%, a higher percentage than the assumed value for euhydrated individuals, which is set as 73.2%. A systematic review showed that preterm newborns present a higher TBW percentage compared to full-term individuals (73.8%). TBW of preterm reached up to 90% at 26 weeks of gestation, dropping to 75% at 36 weeks of gestation, and dropping 1.44% per week after birth (53). Estimation of total body water by the 2H_2_O dilution method from healthy individuals (children to adults) showed that prepubescent children have a higher aqueous fraction of their fat-free body mass when compared to young adults (72.7 ± 1.6% vs. 70.8 ± 1.2%; *p* < 0.01) (54). Lohman et al. described the chemical composition of FFM changes during childhood and they were both ages- and sex-specific (55). Therefore, our data contribute to characterizing the TBW/FFM in VLBW preterm, since there is still a paucity of data in this population during growth ex *utero* (53).

Another interesting finding of early stimulation in the VLBW preterm population was the relationship between visceral fat area and insulin resistance, showing that CG presented higher insulin resistance with more visceral fat, a relation not observed in the IG group. Prematurity has been considered a risk factor for cardiovascular and metabolic diseases (5, 56), in which preterm-born individuals presented a higher incidence of hypertension (57), glucose intolerance (52, 58), as well as metabolic syndromes (59, 60). The inverse association observed in our study agrees with the observation that young adults born extremely preterm present a higher number of risk factors for cardiometabolic disorders unrelated to each other as observed in the control term group (52). Although our results are not strong enough to infer many interpretations, we believe that the protective response observed in IG could be related to the light increase of FFM components, such as skeletal muscle mass, which is insulin sensitive and regulates glucose metabolism, and contributes in the prevention of cardiometabolic disorders (61). Since early stimulation can positively affect the central nervous system, thus improving neurodevelopment, it can also positively affect the other systems, as is observed in exercising: it can improve whole-body glucose tolerance, lipid handling, and insulin sensitivity in humans (62), as well as in rodents (63).

Our follow-up study presents limitations. Although we have been using in our cohort of children born preterm the research grade InBody 770^®^, a multi-frequency BIA that presents more reliable results of water compartments (38), we should be careful with the data interpretation. However, many studies have shown that BIA results correlate well with the gold standard method DEXA scan. This implies that BIA demonstrated a strong accuracy and reliability when compared to DEXA (64, 65) which has been recently used in children of preschool age (66–68). Moreover, the use of this equipment is advantageous for clinical use and large-scale epidemiological studies since it is simple, rapid, non-invasive, accessible, reliable, and requires little training. This will allow an appropriate follow-up of the preterm population growth and development from our cohort (69). A study with the purpose to examine the validity of body composition showed that skeletal muscle mass was reliably measured using a multi-frequency BIA method in preschool children (70). Using BIA in preschool-aged children can be difficult due to the child’s movement during measurement; the evaluation was considered complete if the child was able to stay still for 60 s over the scale holding the electrodes. Further studies from our preterm cohort population and additional follow-up evaluations, at both preschool and school ages, will contribute to confirming these preliminary results. Another limitation of this study was the small sample size due to unreachable subjects, as well as the disinterest of the legal guardians (parents) to participate in the study due to the pandemic and socio-economic-related factors.

## Conclusion

This study was developed to investigate if an early intervention program could positively affect body composition in VLBW preterm children. The intervention indicates that it can probably increase FFM and modify the relationship between fat and the endocrine system, which may contribute to better health with advancing age in VLBW preterm children. Nonetheless, further longitudinal studies and follow-ups of these preterm children are required to establish the clinical significance and prolonged impact of early intervention in this population.

## Data availability statement

The data that support the findings of this study are available on request from the corresponding authors JB, jbernardi@hcpa.edu.br and RF, rolfernandes@hcpa.edu.br. The data are not publicly available due to their containing information that could compromise the privacy of research participants.

## Ethics statement

The ethical approval to conduct this study was granted by the Research Ethics Committee of the Hospital de Clínicas de Porto Alegre (number HCPA: 2019-0809; Certificate of Presentation for Ethical Appreciation (CAAE): 27358019.1.0000.5327). The guardians of the participants who met the eligibility criteria were invited to participate in the study and were included after signing the informed consent form.

## Author contributions

RF and JB conceptualized and designed the study, performed data analyses, and wrote the manuscript. JF and FG participated in data collection and conducted the study. RP and RS conceptualized and designed the study, reviewed critically the manuscript, and obtained funding. All authors revised and finalized the manuscript.
